# Feasibility, acceptability and preliminary clinical outcomes of a brief coping-focused intervention for delusions blended with smartphone-based ecological momentary assessment and intervention in persons with schizophrenia spectrum disorders: A pilot single-arm trial

**DOI:** 10.1016/j.invent.2025.100804

**Published:** 2025-01-27

**Authors:** Kerem Böge, Niklas Bergmann, Inge Hahne, Laura Emde, Sharla Cartner, Imogen Bell, Matthias Pillny, Neil Thomas

**Affiliations:** a Department of Psychiatry and Neuroscience, Campus Benjamin Franklin (CBF), Charité – Universitätsmedizin Berlin, Germany; b Medical University Brandenburg, Department of Psychology, Neuruppin, Germany; c German Center of Mental Health (DZPG), side Berlin/Potsdam; d Centre for Mental Health, Swinburne University of Technology, Melbourne, Australia; e University of Melbourne, Orygen, Melbourne, Australia; f University of Hamburg, Division of Clinical Psychology and Psychotherapy, Germany

**Keywords:** EMA, EMI, Experience sampling method, Coping strategy enhancement, Delusions, Schizophrenia spectrum disorders, Cognitive behavioral therapy for psychosis

## Abstract

Delusions are one of the core symptoms of schizophrenia spectrum disorders (SSD), associated with distress and impaired functioning. Traditional Cognitive Behavioral Therapy (CBT) approaches are less effective for delusions, require significant resources, and specialized staff training. Symptom-specific therapy approaches, which target factors involved in the development and maintenance of psychotic symptoms, provide a valid alternative. Recent research demonstrates the efficacy of coping strategies as an intervention element, however, so far, only in the context of auditory hallucinations. Digital technologies, such as ecological momentary assessment (EMA) and ecological momentary intervention (EMI), are gaining attention in mental health, providing enhanced assessment and intervention opportunities. The present single-arm trial aimed to investigate the feasibility, acceptability, and preliminary outcomes of a smartphone-based blended EMA/I psychological therapy approach focusing on improving coping strategies for delusions in SSD. In total, *N* = 10 participants received four face-to-face therapy sessions alongside German university-level treatment-as-usual over an intervention period of four to six weeks. Feasibility was assessed by completion rates of the EMA/I questionnaires, use of the application between sessions and recruitment rates. Acceptability was assessed by a satisfaction questionnaire, open feedback, and analysis of adverse effects. Clinical outcomes included self-rated and rater-based intensity and distress of delusions and comorbid symptoms at pre- and post-intervention. Findings supported the feasibility and acceptability of the DICE (DICE - Delusion Ideation in the Context of Everyday life intervention) intervention, with high retention (10/13 participants, 77 %) and completion rates for the EMA- (59 %) and EMI-questionnaires (72 %), as well as a high protocol adherence (90–97 %), exceeding all predefined benchmarks. Open feedback indicated good satisfaction, with all participants using the application between sessions, reflecting a high engagement level. Clinical outcomes displayed relevant changes in ameliorating the intensity of delusions when being measured by the Psychotic Symptom Rating Scales as well as by the Green Paranoid Thought Scale, and self-rated improvements in distress and depressive symptoms. Changes in the intensity and distress of delusions might be explained by improved coping behaviour. Further research with control conditions is needed to validate findings and analyze the efficacy as well as mechanisms of actions of the intervention in a fully powered trial.

## Highlights

• Blended-care through EMA/EMI-based approach seems to be feasible and acceptable in persons with psychosis
• The intensity of delusions was reduced at post-intervention, irrespective of the outcome being self-rated or rater-based
• Open feedback showed high satisfaction, usage, and engagement levels
• This EMA/I-based novel therapeutic approach combines cognitive behaviour therapy and symptom-specific strategies

## 1 Introduction

Schizophrenia spectrum disorders (SSD) are characterized by positive symptoms, such as delusions, hallucinations, disorganized thinking and behaviour, and negative symptoms, such as reduced affective resonance, avolition, diminished motivation as well as social activity (e.g. ICD-10: F2X.X) (Graubner and Auhuber, 2021). In particular, delusions, defined as fixed beliefs that are not amenable to change in light of conflicting evidence, (World Health Organization, 2019) appear to be frequently associated with significant distress and impaired social functioning (Lincoln and Heibach, 2017). While cognitive behavioral therapy for psychosis (CBTp) (Farhall and Thomas, 2013; Turkington et al., 2006) is being recommended as a treatment approach for patients with SSD in national (Gaebel et al., 2019) and international guidelines (NICE, 2002, NICE, 2014), the efficacy of CBTp remains small to moderate (Jauhar et al., 2019; Jones et al., 2018). Although CBTp is effective in ameliorating psychotic symptoms, the components that may impact psychotic processes seem to be not activated in unified CBTp approaches targeting overall symptom severity (Lincoln and Peters, 2019). Recently, research interest has shifted towards symptom-specific, individualized treatment approaches (Freeman, 2011; Lincoln and Peters, 2019; Newman-Taylor and Bentall, 2024). Cognitive theories of delusions have emerged to analyze which cognitive processes are associated with the occurrence of delusions (Frith, 2015; Garety and Hemsley, 2013). The goal of these theories is to inform about cognitive and emotional factors which lead to the development and maintenance of delusions and to derive therapeutic interventions, one of which is focusing on coping behaviours (Freeman and Garety, 2014; Rückl et al., 2015).

Research has shown that differences in the individual's ability to cope with psychotic symptoms may play an important role in symptom exacerbation (MacAulay and Cohen, 2013), and the pathogenesis of SSD (Phillips et al., 2009). The theoretical background for this is provided by the stress-appraisal-coping framework (Lazarus and Folkman, 1984), which is an extension of the stress-vulnerability hypothesis of schizophrenia (Zubin and Spring, 1977). Within this framework, coping is defined as a dynamic process that is highly context-dependent and determined by ongoing cognitive appraisals of the stressor, aiming to reduce stress. Until now, the stress-appraisal-coping framework has been successfully applied to hearing voices (Falloon and Talbot, 1981; Farhall, 2005), leading to the “Coping Strategy Enhancement” program by Tarrier and colleagues (CSE; (Tarrier et al., 2009)). The program aims to train existing coping strategies, which builds upon findings from a descriptive study in which the authors found that successfully coping with auditory hallucinations resulted from the use and systematic application of coping strategies (Falloon and Talbot, 1981). Trials on the CSE-approach have supported the efficacy of this program in reducing overall symptom severity (Bell et al., 2020; Hayward et al., 2018; Tarrier et al., 1993; Tarrier et al., 1998; Yusupoff and Tarrier, 2019). Limitations exist regarding its effects on delusions, with just two case studies reporting positive evidence (Tarrier et al., 2009).

Furthermore, the CSE approach builds upon a functional analysis of the targeted symptoms to inform the implementation of effective coping more consistently (Tarrier et al., 2009), therefore relying on idiographic assessment methods to individualize a person's self-management. This can be limited by the person's ability to observe, recall and communicate patterns in symptom variation, which can lead to retrospective recall errors (Schwarz, 2007). Two approaches that can potentially improve the CSE approach are ecological momentary assessment (EMA) (Shiffman et al., 2008) and ecological momentary intervention (EMI) (Heron and Smyth, 2010). The application of digital technologies in the therapy of SSD has the potential to extend the assessment and monitoring of symptoms to the context of daily life (Bell et al., 2024). Furthermore, targeted psychotic symptoms can be addressed directly in their context, for example, by using reminders as a real-time intervention, which also fosters the translation of learned skills to real-life (Bell et al., 2020; Ben-Zeev et al., 2014; Moore et al., 2020). Against the myth that people with SSD are not able to benefit from the use of digital technology for mental health, a growing body of evidence has shown that the use of EMA/EMI in patients with psychotic symptoms is acceptable, feasible, effective and incremental over retrospective reports (Bell et al., 2024; Bell et al., 2020; Ben-Zeev et al., 2011; Depp et al., 2010; Granholm et al., 2012; Hanssen et al., 2020; Moitra et al., 2017), especially when being combined with face-to-face therapy in a blended approach (Erbe et al., 2017). With regards to cognitive processes associated with the occurrence of delusions, Garety and colleagues decided to address the process of reasoning in paranoia and tested a digitally supported reasoning intervention (SlowMo), whereby no significant improvements in the primary measure of paranoia could be demonstrated, however, promising improvements on paranoia outcomes as secondary outcomes (Garety et al., 2021).

The only study to explore the application of EMA/EMI to improve the process of coping with psychotic experiences was targeting auditory hallucinations - another key psychotic symptom dimension in the same category of positive symptoms as delusions. Informed by the CSE framework, Bell and colleagues developed a smartphone-assisted coping-focused intervention SAVVy for hearing voices, which used EMA to assess fluctuations in hearing voices and EMI to support the implementation of personalized coping responses in daily life (Bell et al., 2018). In a pilot randomized controlled trial, with participants allocated to treatment as usual (TAU) + SAVVy compared to TAU, different benchmarks and self-rated as well as rater-based measures were used to assess the feasibility and acceptability of the intervention, with the primary clinical outcome being the total score of the Acoustic Hallucinations (AH) subscale of the Psychotic Symptom Rating Scales (PSYRATS) (Haddock et al., 1999). Findings supported the feasibility and acceptability of the approach. There were moderate between group effects indicating a reduction in overall voice severity, which nearly approached significance despite the small sample size (F (1,31) = 3.00, *p* = .09) (Bell et al., 2020).

Nonetheless, patients reported an improved understanding of their symptoms and overall coping (Bell et al., 2020; Moore et al., 2020). Based on the preliminary findings surrounding the SAVVy intervention and the efficacy of therapeutic interventions targeting delusions remaining limited (van der Gaag et al., 2014), the transfer of a coping-focused intervention blended with EMA/-I into the context of delusions seems promising. The current research project aims to examine the feasibility and acceptability of a novel smartphone-based application combined with blended EMA/-I to foster coping with delusions (DICE). Feasibility and acceptability will be indexed by whether participants use the application between sessions (> 80 % of participants), the target level of EMA-data for analysis (> 80 % of clients provide EMA-data), the completion rates of the EMA questionnaires (minimum threshold of >33 % of completed EMA questionnaires) and the rate of intervention completion (>80 % complete all sessions). Furthermore, possible changes in the intensity and distress of delusions, depression, anxiety, stress and the process of recovery are examined.

## 2 Methods

### 2.1 Design

A single-arm open-label feasibility trial was conducted to evaluate feasibility and acceptability of the DICE intervention in participants with SSD experiencing delusions. Following the initial screening for eligibility, participants provided their informed consent. All participants received four face-to-face therapy sessions with a psychotherapist in training while using a smartphone application between sessions. In addition, participants continued to receive their treatment as usual (TAU). Rater- and self-reported assessments were completed at baseline (T_0_) and after four-six weeks post-intervention (T_1_). Participants received 50 Euro as financial compensation after completion. The study was conducted in accordance with the ethical committee of the Charité – Universitätsmedizin Berlin (EA1/257/22) and was registered on clinicaltrials.gov (NCT06207526).

### 2.2 Inclusion and exclusion criteria

*Inclusion* criteria were: 1) being between 18 and 65 years of age; 2) proficiency in the German language; 3) fulfilling the diagnostic criteria for SSD as determined by the Mini-International Neuropsychiatric Interview (M.I.N.I.) (Sheehan et al., 1998); 4) experiencing delusions and significant self-reported distress as assessed by the PSYRATS Delusions (Haddock et al., 1999), Green Paranoid Thoughts Scale (Freeman et al., 2021), and Peters Delusion Inventory (Peters et al., 2004); 5) no recent (past 4 weeks) or planned change in antipsychotic and other psychopharmacological medication; 6) familiarity with the use of a smartphone or being willing to learn how to use one; 7) time availability to attend four psychotherapy appointments and two rating appointments. *Exclusion* criteria were: 1) severe visual impairment; 2) acute suicidality; 3) pronounced delusional symptoms (corresponds to a total score > 25; persecution: > 28 in the Green Paranoid Thoughts Scale (Freeman et al., 2021) and 4) acute substance abuse apart from nicotine.

### 2.3 Intervention

**Fig. 1:**
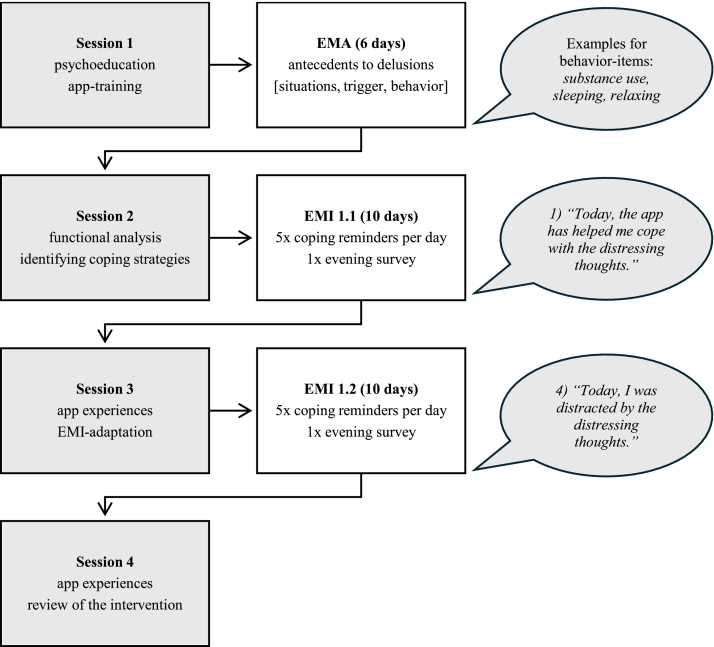
Overview of the intervention phases. Note. EMA = Ecological momentary assessment; EMI = Ecological momentary intervention.

The intervention is based on an intervention by Bell et al. that has demonstrated good feasibility, acceptability, and preliminary effectiveness for auditory verbal hallucinations (Bell et al., 2018; Bell et al., 2020). The present intervention was thematically adapted to the context of delusions and used the mPath app (KU Leuven) (Mestdagh et al., 2023). An overview of the intervention phases is depicted in Fig. 1. The intervention was divided into two phases: functional assessment of the symptoms and applying/practicing coping strategies. After baseline assessment, which was followed by a ten minute app training with a research assistant, participants took part in the first therapy session, which focused on psychoeducation and the preparation of the functional analysis. Following the first session, participants completed six days of EMA monitoring. This involved completing ten surveys daily, focusing on momentary fluctuations of their delusions. The survey consisted of 39-EMA-items and was specifically created for the purpose of this study. The development of the items involved several iterative stages, including consultations with the experts of the SAVVy-trial, resulting in items which measure common antecedents to delusions, delusion-related variables in terms of frequency and content, as well as coping behaviours to the delusions. Analysis of the EMA data, using multiple regressions, produced a personalized template indicating variables associated with fluctuations in delusions intensity. This was then used in the second phase of the intervention to inform the formulation of coping strategies aiming to reduce intensity, distress and impact of the delusions. Together with the patient, three individualized coping strategies were selected and programmed into the app. Thereafter, participants received five coping reminders and an eight-item survey each day, monitoring their delusions and assessing the helpfulness of the coping strategies, in each case for ten days after therapy sessions two and three. The therapy sessions were used to discuss experiences with the app and allowed changes to be made to the personalized EMI. The intervention ended with a final session involving a review of the intervention.

Participants were recruited in the outpatient facility at the Charité – Universitätsmedizin Berlin, Campus Benjamin Franklin, as well as via advertising to outpatient services in Berlin, such as counseling centers and assisted living facilities. Participants continued their outpatient treatment. At the outpatient facility of the Department of Psychiatry, Charité, CBF, routine care includes monthly consultations with a psychiatrist, psychotherapeutic sessions once a month by a psychotherapist in training, pharmacological treatment and support from social workers. In accordance with official S3 national guidelines, psychiatrists will provide evidence-based psychopharmacological treatment within the recommended therapeutic range, whereby we included the medication regime in our demographic surveys. The individual TAU was assessed at T_0_ and T_1_. This included contact with social psychiatric services and psychiatric contact. Additional psychotherapy was quantified in frequency and duration.

### 2.4 Assessment

#### 2.4.1 Feasibility and acceptability

As a pilot study, the primary outcomes were acceptability and feasibility. Feasibility was operationalized with an approach of using traffic-light benchmarks (stop (< 60 %), adjust (≥ 60 % < 80 %), and go (>80 %)) to clearly define thresholds for the outcomes, by using progression criteria's (Avery et al., 2017). The assessment contained the usage registration in percentage, by whether participants used the application between sessions (benchmark: > 80 % of participants), the target level of EMA-data for analysis (benchmark: > 80 % of clients provide EMA-data) and the completion rates of the EMA questionnaires (minimum threshold of >33 % of completed EMA questionnaires).

Acceptability was assessed by using the DICE intervention checklist (Appendix A), in which the two psychotherapists in training who administered the intervention rated the engagement of the participant (range 1–5) and the protocol adherence (0–100 %) at the end of each therapy session. Additionally, a series of open-ended questions about elements of the intervention were assessed. Side-effects and adverse events were recorded using the Negative Incidents and Effects Questionnaire (Rozental et al., 2019).

#### 2.4.2 Rater-based and subjective intensity of delusions

The psychotic symptom rating scale, PSYRATS (Haddock et al., 1999), consists of two subscales, one to assess auditory hallucinations and the second to assess delusions, both designed as semi-structured interviews. The mean values of the delusion subscale were calculated to operationalize the objective intensity of delusions. The Green Paranoid Thought Scale in its revised form, R-GPTS (Freeman et al., 2021), was used to record the subjective intensity of delusions. The instrument consists of two separate subscales, with eight items assessing ideas of reference and ten items assessing ideas of persecution, currently being the best measure of paranoid thoughts (Statham et al., 2019). Higher sum scores indicate greater levels of paranoid thoughts. While paranoia is relevant and the most dominant theme of delusional thinking in clinical groups (Collin et al., 2023; Freeman et al., 2021) it still only represents a subset of delusional themes. Therefore the Peters Delusion Inventory (PDI, (Peters et al., 2004)) was included as a second outcome parameter to assess the subjective intensity of delusions. The PDI has the advantage of capturing the multidimensional nature of delusional ideation. Five scores can be obtained from the items: a dichotomous score, indicating the presence or non-existence of a delusional theme, a distress score, a preoccupation score, a conviction score, and a total score. Only the latter is useful as a global measure of delusional ideation and requires adding up the four previous scores, with higher scores indicating greater levels of delusional ideation.

#### 2.4.3 Psychological distress

Since the PDI records delusions multidimensional, the distress score was used separately as an outcome parameter. The distress score adds discriminative power to the PDI total score in distinguishing individuals with psychotic symptoms from controls (Peters et al., 1999; Sisti et al., 2012), therefore qualifying as a parameter to assess psychological distress associated with experiencing delusions.

#### 2.4.4 Further outcomes

Self-reported questionnaires were administered to assess depression, anxiety, stress (Depression Anxiety Stress Scale; DASS (Nilges and Essau, 2015)) and the process of recovery (Questionnaire about the Process of Recovery; QPR (Neil et al., 2009)).

### 2.5 Data management

All data collection and management were conducted using electronic case report file (eCRF) programmed with the study software REDCap (Harris et al., 2019). Study personnel involved in the data assessment and management received structured training. The eCRF software holds an authentication procedure with individualized role management and safe and encoded connections. All data were stored on secure data servers hosted by the Charité – Universitätsmedizin Berlin.

### 2.6 Statistical analyses

All analyses were conducted with RStudio Version 2022.12.0 + 353 (Posit Software, 2022). As this is a pilot single-arm trial evaluating the feasibility and acceptability of intervention, power calculations based on effect sizes are not appropriate. As one goal of the pilot study is to gather preliminary data of outcome measures, which can be used to conduct a sample size calculation for a larger follow-up trial, we followed the recommendations on sample sizes for pilot studies, which vary between 10 and 30 participants per trial arm (Hertzog, 2008; Lancaster et al., 2004).

Demographic statistics were summarized as frequencies for categorical variables and means and standard deviations for continuous variables. Feasibility and acceptability results were reported descriptively according to the defined benchmarks (stop (< 60 %), adjust (≥ 60 % < 80 %), and go (>80 %)) for further progression of the approach (Avery et al., 2017). Secondary outcome measures were summarized by means and standard deviations. For the analysis of effects on intensity and distress of delusions as well as comorbid symptoms, within-group changes for DICE + TAU were conducted by comparing pre- to post-intervention levels using paired *t*-tests. The significance level was set at 0.05, and *p*-values were reported for one-sided testing. 95 % Confidence Interval of the mean difference score (T_1_ – T_0_) were being calculated. The classification of the effect sizes was based on the conventions of Cohen, with *d* = 0.2 being considered a small effect size, *d* = 0.5 representing a medium effect size and *d* = 0.8 representing a large effect size (Cohen, 2013).

## 3 Results

### 3.1 Sample characteristics

**Fig. 2:**
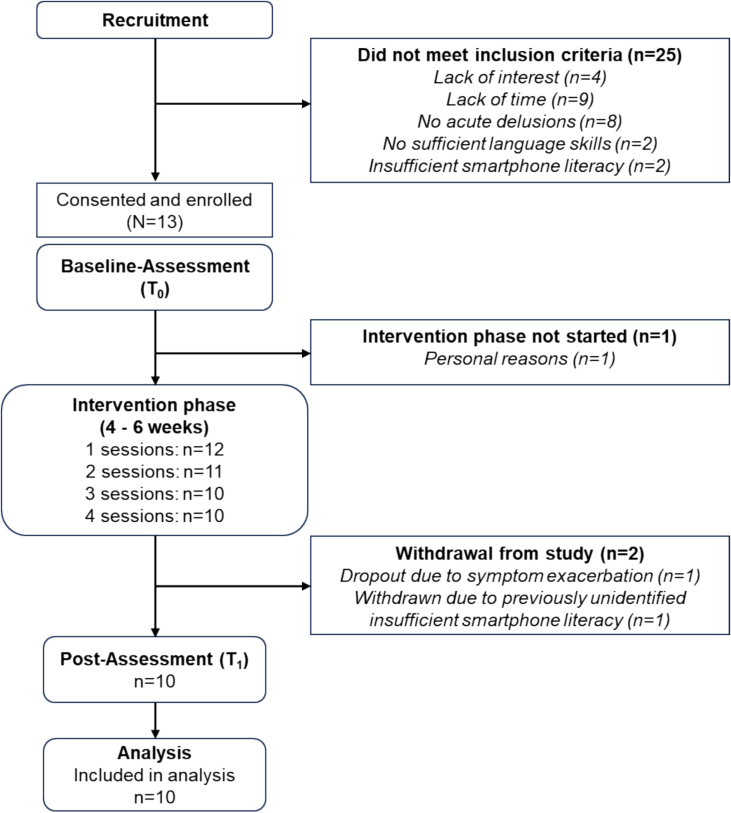
DICE Flow diagram. Note. Adaptation of a standard CONSORT Flow diagram.

In total, thirty-eight participants were screened for eligibility. Overall, there was a 34 % uptake in the trial. Thirteen participants were included in the study, of which one participant dropped out before the intervention phase started due to time restrictions. One participant dropped out after session two due to an exacerbation of symptoms. This patient's treatment appointments were delayed due to an infection and he perceived the large time gap which resulted between appointments as a burden. Another patient was excluded during the intervention phase as it turned out that he did not meet the smartphone literacy criterion. The final sample consisted of *N* = 10 participants. An overview of the Consolidated Standards of Reporting Trials (CONSORT) flow diagram is illustrated in Fig. 2. Most participants identified as male (80 %), native German speakers (60 %), and highly educated (70 % ≥ A levels). The duration of illness in years was *M* = 11.40 (*SD* = 6.52), indicating a chronic course on average. Differences in the medication type, number, and dose equivalent between T_0_ and T_1_ were non-significant, *t*(9) = 0.14, *p* = .890. A detailed description of sociodemographic characteristics, types of delusions and medication regimes are displayed in Table 1, Table 2, respectively.

**Table 1:** Sociodemographic variables at baseline.

Variable	DICE + TAU
	n / mean (SD)
Sex	
Male	8
Female	2
Age	39.00 (9.30)
Nationality	
German	6
American	1
Japanese	1
Belarusian	1
Latvian	1
Family Status	
Single	7
Divorced	2
In a relationship	1
Current housing situation	
Private flat (alone)	7
Assisted living	2
Other	1
Highest educational achievement	
Higher secondary school or less	3
A level	4
Diplom	1
Master's degree	2
Occupation	
Employed (full-time)	1
Employed (part-time)	5
Student	1
Unemployed	3
Years of Illness	11.40 (6.52)
Type of delusion	
Double meanings	6
Personal messages in magazines/television	5
Perceiving people as not who they appear to be	4
Being pursued	6
Sense of a conspiracy	4
Destined to be someone important	4
The feeling of being a special person	6
Close connection to God	6
Telepathy	4
Interference from electrical devices	3
Chosen by God	2
Witchcraft, voodoo and the occult	4
Infidelity of the partner	2
Being more sinful than others	3
Being perceived as strange	4
Feeling of having no thoughts	3
Doomsday feelings	5
Strange thoughts	6
Very vivid and lively thoughts	4
Echoing back of thoughts	2
The feeling of being a robot or zombie	2

Notes. N = 10; DICE = delusion-focused intervention; TAU = treatment-as-usual.

**Table 2:** Medication regime at baseline and post-intervention.

Type, number and dose equivalent of medication	Baseline	Post-intervention		
	DICE + TAU	DICE + TAU	*t*	*p*
	n / *mean*	n / *mean*		
Antipsychotics				
0	2	2		
1	5	5		
2	1	1		
≥ 3	2	2		
Olanzapine equivalent dose in mg^a^	20.38	20.34	0.14	0.89
Antidepressants				
0	5	5		
1	4	4		
2	0	0		
≥ 3	1	1		
Mood stabilizers				
0	9	9		
1	1	1		
Benzodiazepines				
0	9	9		
1	1	1		

Notes. Numbers refer to the n of participants in each group receiving the corresponding number of medications at the time-points. There were no differences in the sociodemographic variables besides medication regime. P-value is based on a two-sided t-test for continuous variables; DICE = delusion-focused intervention; TAU = treatment as usual.

a Dosages were converted to Olanzapine (Leucht et al., 2015).

### 3.2 Feasibility and acceptability

The study retention rate was 10/13 (77 %). One participant dropped out after inclusion, before the intervention phase started, due to time restrictions. Two participants were withdrawing during the intervention. These withdrawals were due to one participant dropping out after session two and one participant which had to be withdrawn due to previously unidentified insufficient smartphone literacy. The average completion rate of the EMA questionnaires was 59 %; all participants exceeded the 33 % completion rate criteria, indicating the feasibility of integrating EMA into the intervention. All participants (benchmark: > 80 % of clients) used the applications between the sessions. The average EMI completion rate was 72 %. Engagement during each session, being rater-based assessed in the DICE intervention checklist (range 1–5), revealed overall high engagement across the four therapy sessions, *M*(session 1) = 4.65; *M*(session 2) = 4.50; *M*(session 3) = 4.50; *M*(session 4) = 4.70. Protocol adherence averaged 90–97 %, across sessions. Responses to open-ended questions at post-intervention reflected mainly positive feedback, indicating high acceptability of DICE. All points of feedback are displayed in Table 3.

**Table 3:** Open feedback from participants regarding the application of the app.

Participant #1:	• Pro: Easy to use, constant companion who always reminded you to use coping strategies • Con: Evening survey was too long
Participant #2:	• Pro: Builds upon existing coping strategies. Helps you to better recognize your own state of mind • Con: Signals sometimes appeared at inappropriate moments, evening surveys were sometimes very late
Participant #3:	• Pro: The approach made it possible to individualize the therapy • Con: /
Participant #4:	• Pro: Therapy and app worked hand in hand, helpful addition to the therapy • Con: Overwhelming at first
Participant #5:	• Pro: Strategies were made more present in daily life • Con: Too much text, too many EMA surveys
Participant #6:	• Pro: Insights from the meeting were reactivated, assistance with coping • Con: Reminders were not always needed
Participant #7:	• Pro: Reminders, helpful to talk about the content of the delusions • Con: Handling of the app (too much scrolling down)
Participant #8:	• Pro: More self-reflection, more comprehensive support through the app • Con: /
Participant #9:	• Pro: Transfer of therapy into daily life • Con: /
Participant #10:	• Pro: Enhanced training of coping • Con: Limited to three strategies

It should be emphasized that in the current study, only one participant dropped out during the intervention phase due to an exacerbation of his symptoms, classed as an unrelated serious adverse event (SAE) according to the German SAE Report Form for reporting of SAE in clinical trials or performance evaluation (§ 3 (5) of the Ordinance on Medical Devices Vigilance).

### 3.3 Preliminary clinical outcomes

Regarding the ownership of digital devices, all participants stated that they owned mobile devices, predominantly smartphones. Furthermore, all participants self-reported confidence in using and accessing the internet and mobile devices (yes/no-item: “Do you feel confident using the Internet, e.g. when logging in and navigating websites?”). In terms of frequency, the sample predominantly stated that they always have access to the internet (once a day: 1; more than once a day: 1; always: 8) and use it frequently (a few times a week: 1; once a day: 1; more than once a day: 5; always: 3). Table 4 in the Appendix (B) displays all frequencies of the screened skills in the use of and access to the internet.

**Fig. 3:**
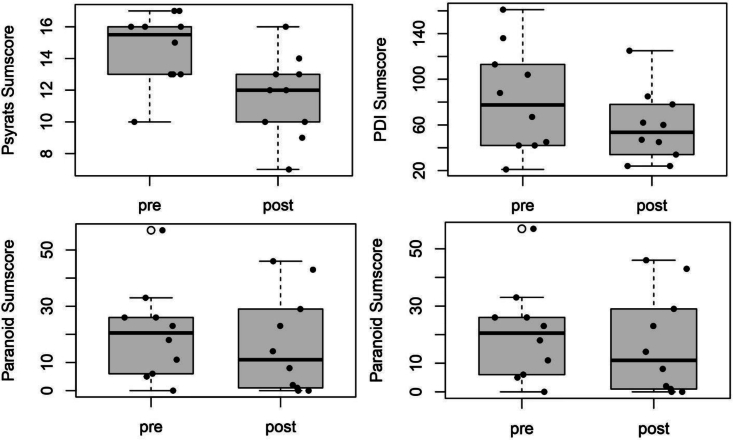
Boxplots of the sum scores of intensity/distress of delusions and intensity of paranoid delusions before and after the intervention. Notes. From left to right: a) Sumscores of the intensity of delusions, measured rater-based; b) Sumscores of the intensity of delusions, measured subjectively; c) Sumscores of the distress of delusions; d) Sumscores of the intensity of paranoid delusions. Separate analyses without the outliers were not carried out, as the outlier values were clinically justifiable.

The estimated means of the PSYRATS–delusion score, as a rater-based measure of the intensity of delusions, indicated a lower mean intensity of delusions at post-intervention, *M* = 11.60, *SD* = 2.63, compared to pre-intervention, *M* = 14.60, *SD* = 2.27. Paired *t*-tests revealed that participants significantly improved from pre-intervention to post-intervention; *t*(9) = 4.88, *p* < .001, *d* = 1.54. There was an improvement regarding the subjective intensity of delusions (PDI-total score), with participants showing lower mean values at post-intervention (*M* = 58.40, *SD* = 31.30) compared to pre-intervention (*M* = 81.90, *SD* = 46.13). This difference reached conventional levels of statistical significance, *t*(9) = 1.86, *p* = .048, *d* = 0.59. Participants showed descriptive differences in intensity levels of paranoid delusions from pre- to post-intervention. Paired *t*-testing indicated a moderate effect size, d = 0.44, which did not reach statistical significance, *t*(9) = 1.38, *p* = .100. A paired t-test was performed to evaluate whether the participants' distress related to the delusions decreased from pre-intervention (*M* = 25.60, *SD* = 13.59) to post-intervention (*M* = 18.30, *SD* = 9.60). The results indicated that the difference was significant, PDI-distress scores: *t*(9) = 2.05, *p* = .036, *d* = 0.65. Fig. 3 displays these results illustrated as boxplots of the sum scores at pre and post-intervention.

Regarding comorbid symptoms, significant improvements were observed in depressive symptoms (t(9) = 2.13, *p* = .031, d = 0.67) but not in anxiety and stress symptoms. Participants showed descriptive differences in their recovery process from pre- to post-intervention. Paired t-testing did not reach statistical significance (t(9) = −1.64, *p* = .068, d = −0.52). An overview of all paired *t*-tests can be seen in Table 5.

**Table 5:** Means, standard deviations and within-group changes for all clinical outcomes.

Scales and subscales	T_0_	T_1_	Within-group changes (T_0_ to T_1_)				
	DICE + TAU						
	*mean* (*SD*)	*mean* (*SD*)	95 % CI	*t*	*p*	Δ	*d*
PSYRATS *– delusion*	14.60 (2.27)	11.60 (2.63)	0.69; 5.31	4.88	< 0.001	3.00	1.54
PDI	81.90 (46.13)	58.40 (31.30)	−13.90; 60.90	1.86	0.048	23.50	0.59
*- yes/no score*	8.50 (4.28)	6.40 (3.34)	−1.52; 5.72	1.73	0.059	2.10	0.55
*- distress score*	25.60 (13.59)	18.30 (9.60)	−3.85; 18.45	2.05	0.036	7.30	0.65
*- preoccupation score*	24.20 (14.33)	17.10 (9.85)	−4.58; 18.76	1.70	0.061	7.10	0.54
*- conviction score*	23.60 (14.45)	16.60 (9.23)	−4.54; 18.54	1.78	0.055	7.00	0.56
R-GPTS	20.50 (16.74)	16.60 (17.78)	−12.33; 20.31	1.38	0.101	3.90	0.44
*- ideas of reference*	9.10 (6.19)	7.90 (8.27)	−5.70; 8.10	0.99	0.174	1.20	0.31
*- ideas of persecution*	11.40 (10.93)	8.70 (9.96)	−7.13; 12.53	1.28	0.117	2.70	0.40
DASS	22.00 (8.73)	16.70 (8.04)	−2.59; 13.19	1.99	0.039	5.30	0.63
*- depression*	8.80 (4.42)	6.10 (3.60)	−1.10; 6.50	2.14	0.031	2.70	0.67
*- anxiety*	5.60 (2.63)	4.60 (2.41)	−1.37; 3.37	1.25	0.122	1.00	0.39
*- stress*	7.60 (3.57)	6.00 (3.74)	−1.83; 5.03	1.60	0.072	1.60	0.50
QPR	36.10 (6.39)	38.70 (8.66)	−9.79; 4.59	−1.64	0.068	−2.60	−0.52

Notes*.* P-values are based on one-sided paired t-tests. DICE = delusion-focused intervention; TAU = treatment-as-usual; SD = standard deviation; PDI = Peters Delusion Inventory; R-GPTS = Revised Green Paranoid Thought Scale; DASS = Depression Anxiety Stress Scales; QPR = Questionnaire about the Process of Recovery; PSYRATS = Psychotic Symptom Rating Scales, T_0_ = pre-intervention; T_1_ = post-intervention; CI = 95 % Confidence Interval of the mean difference scores; Δ = mean difference score (T_1_ – T_0_); *d* = Cohen's d.

## 4 Discussion

The present study examined the feasibility and acceptability of the adapted SAVVy-intervention (Bell et al., 2018; Bell et al., 2020), a blended smartphone-based ecological momentary assessment and intervention approach to foster coping behaviour, newly applied to delusions in outpatients with SSD (DICE). Furthermore, the study aimed to assess the potential clinical effects of the intervention by providing preliminary outcomes on the intensity and distress of delusions, as well as depression, anxiety, stress and process of recovery. Overall, the results support the application, with the first evidence of reducing the intensity and distress of delusions and improvements in depressive symptoms. The assessment of the medication regime at baseline and post-intervention showed no differences in number and doses, as well as stable medication prior to participation (e.g. for four weeks), ruling out an effect based purely on the medication.

Completion rates of the EMA (59 %) and EMI (72 %) questionnaires, usage of the application between sessions (100 % of clients), and the target level of data (100 % of clients) were above the defined benchmarks for progressing with the study (Avery et al., 2017). All participants (100 %) met the minimum 33 % completion rate required to generate the EMA-derived feedback.

The rates were of similar order of magnitude as in the original SAVVy-approach, therefore supporting the feasibility of this approach across populations with positive psychotic symptoms. Especially, the high response rate of EMA and EMI is consistent with other studies investigating digital interventions for individuals with psychotic symptoms in the format of mobile interventions (Bell et al., 2020; Depp et al., 2010; Hanssen et al., 2020; Taylor et al., 2022), supporting the growing evidence that people with SSD participate in similar intensity with digital technologies as other clinical populations (Eisner et al., 2023; Gay et al., 2016). Even though reminder engagement differed significantly between the participants, open feedback indicated the reminders' usefulness. Hence, the lower rates are more likely due to the voluntary nature of the offer. One possible mechanism behind the usefulness of personalized reminders is that they may support the advantages of EMI in fostering the translation of learned skills to real life (Moore et al., 2020), thereby augmenting the intervention (Heron and Smyth, 2010).

Participants generally reported positive open feedback (see Table 3), and the results of the intervention checklist indicate the high acceptability of the intervention. Reasons for non-engagement included concentration issues, forgetting working materials and the length of EMA/-I questionnaires. Protocol adherence in each session was high, with over 85 %, whereby organizational issues, time restructuring (e.g. due to patients being late) and the discussion of problems apart from experiencing delusions caused digressions from the protocol. In the open feedback, one patient emphasized as a strength of the study that it reinforced already functional strategies (see Participant #2; Table 4). As predicted in previous research (Estroff, 1989; Garcelán and Rodríguez, 2002; Strous et al., 2005), EMA analyses pointed out that most participants had already attempted to functionally cope with their symptoms, reflected in the frequency of coping strategies surveyed. As the use of coping strategies is directly associated with the symptomatic manifestation of psychotic symptoms (Meyer, 2001) and functioning (Godoy Izquierdo et al., 2021), the changes in intensity and distress of delusions possibly occur via the proposed mechanism of action by improving coping. While this study was not planned to test mediating or mechanism effects, open feedback regarding the intervention provides tentative evidence for this mechanism of the intervention. The findings regarding reduced distress levels through CSE approaches are limited, preliminary and restricted to auditory hallucinations (Bell et al., 2020; Zanello et al., 2023).

Only one participant dropped out of the study due to an exacerbation of his symptoms, resulting in an unrelated SAE. This finding further underpins a recently published analysis of adverse events reported in digital health interventions (DIH) evaluations for psychosis, supporting the safety of DIH for psychosis, with just 2.7 % of adverse events being related to the DIH, mostly resulting in negative consequences such as affective exacerbation, psychosis exacerbation, or participants stopping the use of prescribed medication (Allan et al., 2024).

Regarding delusions, a previously conducted blended smartphone-based intervention for persecutory delusions based on training thinking skills showed positive effects on delusion severity (Garety et al., 2021). The DICE intervention differs from that intervention by focusing on enhancing coping strategies and self-train alternative responses to delusions to reduce associated distress. For future research using coping-focused interventions, the individual coping ability at baseline might be quantified and included as a covariate, as this plays a vital role in symptom exacerbation (MacAulay and Cohen, 2013), mainly since acute symptoms are usually associated with deficits in coping ability (Ristner and Ratner, 2006).

The current pilot study was the first attempt to apply the SAVVy approach (Bell et al., 2018; Bell et al., 2020) to delusions. Its focus was to explore the feasibility and applicability of the DICE intervention. Consequently, preliminary clinical effects must be discussed in light of limited power and a small sample size. Nevertheless, the estimated means of the PSYRATS delusion score, the PDI score and the depression score indicated statistically significant improvements from pre- to post-intervention (*d* = 0.59–1.54). In line with research supporting symptom-specific, individually tailored therapy approaches (Lincoln and Peters, 2019; van der Gaag et al., 2014), the effectiveness of the treatment approach used in the present study may be derived from focusing directly on the maintenance of delusions rather than targeting the overall cognitive perspective of psychotic symptoms. The DICE intervention is, therefore, a novel approach that uses the synergies between CBTp and symptom-specific therapies. Precisely, the preliminary finding that participants showed reduced intensity of delusions post-intervention expands the application possibilities of coping as a mechanism of symptom change (Schlier et al., 2020) from negative symptoms (Schlier et al., 2024) also to positive symptoms.

Limitations of the study are the single-arm feasibility pilot design and limiting statements regarding the effectiveness of the intervention since no placebo or reference treatment was used for comparison (Cucherat et al., 2020). As some participants had access to psychotherapy sessions as part of their TAU, inferences regarding the changes in symptoms solely due to the DICE intervention are limited and needs to be interpreted with caution.

In addition, the preliminary outcomes must be considered in light of the small sample size, as effect sizes in small studies are more likely to be highly variable and rather inflated (Hackshaw, 2008). Contrary to hypothesized, the subjective assessment of the intensity of paranoid delusions, assessed by the R-GPTS, showed only small reductions from pre- to post-intervention. The R-GPTs values had a large variance, which could be due to the sample size, and as the variance influences the information quality of statistical significance, a high variance makes it difficult to find significant differences (Wasserstein and Lazar, 2016).

Nevertheless, a certain homogeneity is noticeable when looking at the sample. The majority of the sample consisted of male individuals who were highly educated, familiar with digital technology, and chronic in terms of the duration of their illness over the years. This limits the generalizability of the findings. Previous research has been able to show that, in individuals with psychosis, higher education and integration of technology into daily life correlate with higher levels of engagement in online interventions (Arnold et al., 2021; Arnold et al., 2019). A key question that should be investigated in future research is the factors that influence engagement with the intervention, specify the scope of the intervention, and develop potential strategies to remove the barriers to engagement with digital technologies in individuals with SSD.

The present study is the first to investigate the effects of a blended smartphone-based EMA/I approach in improving coping strategies adapted to delusions to reduce the intensity and distress of delusions. Findings suggest high feasibility and acceptability rates of the proposed EMA/I-based intervention in a chronic SSD sample experiencing distressing delusions. Furthermore, preliminary clinical outcomes indicated moderate to large improvements in distress and intensity levels of delusions, as well as depressive symptoms after completing the coping-focused intervention approach and thus represent a valuable addition to the debate around worthy targets in the development of interventions for delusions (Freeman, 2011; Rückl et al., 2015). Chronic symptoms in psychotic disorder are not isolated cases (Fusar-Poli et al., 2017), and in some patients, the treatment of positive symptoms with antipsychotic medications turns out to be ineffective (Leucht et al., 2009) with delusions persisting (Appelbaum et al., 2004), showcasing the need for innovative, new treatment approaches like DICE. A larger and fully powered trial with a control condition is needed to replicate present findings and investigate this intervention's efficacy and mechanisms of actions.

## CRediT authorship contribution statement

KB: conceptualization, funding acquisition, methodology, project administration, resources, writing, supervision. NB: investigation, treatment, project administration, writing. IH: investigation, treatment, review and editing. LE: investigation, formal analysis, writing. SC: methodology, formal analysis, review and editing. IB: conceptualization, methodology, review and editing. MP: review and editing. NT: conceptualization, methodology, funding acquisition, project administration, supervision, review and editing.

## Funding

A grant from the Berlin University Alliance (BUA) and the 10.13039/501100001655German Academic Exchange Service (DAAD) supported this work.

## Declaration of competing interest

NT wrote psychological therapy books on CBT for psychosis. KB wrote psychological therapy books on CBT for psychosis and is co-founder of healthcare start-ups developing digital psychological interventions for psychosis.

## Figures and Tables

**Table:** 

Introduction	
Overview of therapy	
Information about voices	
Recording voice experiences in workbook	
Smartphone app training	

**Table:** 

1	2	3	4	5
Not at all	Minimally	Moderately	Mostly	Fully

**Table:** 

Introduction	
Overview of therapy	
Information about voices	
Recording voice experiences in workbook	
Smartphone app training	

**Table:** 

1	2	3	4	5
Not at all	Minimally	Moderately	Mostly	Fully

**Table:** 

Introduction	
Overview of therapy	
Information about voices	
Recording voice experiences in workbook	
Smartphone app training	

**Table:** 

1	2	3	4	5
Not at all	Minimally	Moderately	Mostly	Fully

**Table:** 

Introduction	
Overview of therapy	
Information about voices	
Recording voice experiences in workbook	
Smartphone app training	

**Table:** 

1	2	3	4	5
Not at all	Minimally	Moderately	Mostly	Fully

**Table 4:** Use of and access to the internet.

Variable	DICE + TAU n
Access to mobile devices *- Phone* *- Computer* *- Tablet* Confident in use and access Access to the internet *- Seldom or never* *- Every few weeks* *- A few times a week* *- Once a day* *- More than once a day* *- Always* Use of the internet *- Seldom or never* *- Every few weeks* *- A few times a week* *- Once a day* *- More than once a day* *- Always*	10 9 4 10 0 0 0 1 1 8 0 0 1 1 5 3

*Notes. N = 10;* DICE = intervention; TAU = treatment-as-usual; n = frequencies.

## Data Availability

The data supporting this study's findings are available from the corresponding author upon reasonable request.

## Appendix A DICE Intervention Checklist

**DICE Intervention Checklist – Session 1**

What was the length of this session in minutes? ___________________________________________

The following were completed in session 1 (tick):

Please record any issues raised in this session below in terms of the content, timing, and any comments made by the participant:

__________________________________________________________________________________

__________________________________________________________________________________

__________________________________________________________________________________

__________________________________________________________________________________

Please estimate the percentage of time over this entire session that you spent on the protocol:

___________%

Please record any deviation from the protocol below (e.g. managing distress, maintaining engagement):

__________________________________________________________________________________

__________________________________________________________________________________

In your opinion, please circle below how engaged the participant was with the intervention protocol during this session:

Record any noted reasons for non-engagement: __________________________________________________________________________________

__________________________________________________________________________________

**DICE Intervention Checklist – Session 2**

What was the length of this session in minutes? ___________________________________________

The following were completed in session 1 (tick):

Please record any issues raised in this session below in terms of the content, timing, and any comments made by the participant:

__________________________________________________________________________________

__________________________________________________________________________________

__________________________________________________________________________________

__________________________________________________________________________________

Please estimate the percentage of time over this entire session that you spent on the protocol:

___________%

Please record any deviation from the protocol below (e.g. managing distress, maintaining engagement):

__________________________________________________________________________________

__________________________________________________________________________________

In your opinion, please circle below how engaged the participant was with the intervention protocol during this session:

Record any noted reasons for non-engagement: __________________________________________________________________________________

__________________________________________________________________________________

**DICE Intervention Checklist – Session 3**

What was the length of this session in minutes? ___________________________________________

The following were completed in session 1 (tick):

Please record any issues raised in this session below in terms of the content, timing, and any comments made by the participant:

__________________________________________________________________________________

__________________________________________________________________________________

__________________________________________________________________________________

__________________________________________________________________________________

Please estimate the percentage of time over this entire session that you spent on the protocol:

___________%

Please record any deviation from the protocol below (e.g. managing distress, maintaining engagement):

__________________________________________________________________________________

__________________________________________________________________________________

In your opinion, please circle below how engaged the participant was with the intervention protocol during this session:

Record any noted reasons for non-engagement: __________________________________________________________________________________

__________________________________________________________________________________

**DICE Intervention Checklist – Session 4**

What was the length of this session in minutes? ___________________________________________

The following were completed in session 1 (tick):

Please record any issues raised in this session below in terms of the content, timing, and any comments made by the participant:

__________________________________________________________________________________

__________________________________________________________________________________

__________________________________________________________________________________

__________________________________________________________________________________

Please estimate the percentage of time over this entire session that you spent on the protocol:

___________%

Please record any deviation from the protocol below (e.g. managing distress, maintaining engagement):

__________________________________________________________________________________

__________________________________________________________________________________

In your opinion, please circle below how engaged the participant was with the intervention protocol during this session:

Record any noted reasons for non-engagement: __________________________________________________________________________________

__________________________________________________________________________________
